# Methods to adjust for multiple comparisons in the analysis and sample size calculation of randomised controlled trials with multiple primary outcomes

**DOI:** 10.1186/s12874-019-0754-4

**Published:** 2019-06-21

**Authors:** Victoria Vickerstaff, Rumana Z. Omar, Gareth Ambler

**Affiliations:** 1 Marie Curie Palliative Care Research Department, Division of Psychiatry, University College London, Gower Street, London, WC1E 6BT UK; 2 Department of Statistical Science, University College London, Gower Street, London, WC1E 6BT UK

**Keywords:** Multiple comparison methods, Multiple outcome, Sample size, Statistical analysis, Randomised controlled trials

## Abstract

**Background:**

Multiple primary outcomes may be specified in randomised controlled trials (RCTs). When analysing multiple outcomes it’s important to control the family wise error rate (FWER). A popular approach to do this is to adjust the *p*-values corresponding to each statistical test used to investigate the intervention effects by using the Bonferroni correction. It’s also important to consider the power of the trial to detect true intervention effects. In the context of multiple outcomes, depending on the clinical objective, the power can be defined as: *‘disjunctive power’*, the probability of detecting at least one true intervention effect across all the outcomes or ‘*marginal power’* the probability of finding a true intervention effect on a nominated outcome.

We provide practical recommendations on which method may be used to adjust for multiple comparisons in the sample size calculation and the analysis of RCTs with multiple primary outcomes. We also discuss the implications on the sample size for obtaining 90% disjunctive power and 90% marginal power.

**Methods:**

We use simulation studies to investigate the disjunctive power, marginal power and FWER obtained after applying Bonferroni, Holm, Hochberg, Dubey/Armitage-Parmar and Stepdown-minP adjustment methods. Different simulation scenarios were constructed by varying the number of outcomes, degree of correlation between the outcomes, intervention effect sizes and proportion of missing data.

**Results:**

The Bonferroni and Holm methods provide the same disjunctive power. The Hochberg and Hommel methods provide power gains for the analysis, albeit small, in comparison to the Bonferroni method. The Stepdown-minP procedure performs well for complete data. However, it removes participants with missing values prior to the analysis resulting in a loss of power when there are missing data. The sample size requirement to achieve the desired disjunctive power may be smaller than that required to achieve the desired marginal power. The choice between whether to specify a disjunctive or marginal power should depend on the clincial objective.

**Electronic supplementary material:**

The online version of this article (10.1186/s12874-019-0754-4) contains supplementary material, which is available to authorized users.

## Background

Multiple primary outcomes may be specified in a randomised controlled trial (RCT) when it is not possible to use a single outcome to fully characterise the effect of an intervention on a disease process [1–3]. The use of multiple primary outcomes (or ‘endpoints’) is becoming increasingly common in RCTs. For example, a third of neurology and psychiatry trials use multiple primary outcomes [4]. Data on two primary outcomes (abstinence and time to dropout from treatment) were collected in a trial evaluating the effectiveness of a behavioural intervention for substance abuse [5] and data on four primary outcomes were collected in a trial evaluating a multidisciplinary intervention in patients following a stroke [6]. Typically, these outcomes are correlated and often one or more of the outcomes has missing values.

Typically multiple statistical tests are performed to investigate the effectiveness of the intervention on each outcome. If two outcomes are analysed independently of each other at the nominal significance level of 0.05, then the probability of finding at least one false positive significant results increases to 0.098. This probability is known as the familywise error rate, ‘FWER’. One approach to control the FWER to its desired level is to adjust the *p*-values corresponding to each statistical test used to investigate the intervention effects. Many adjustments have been proposed including the Bonferroni [7], Holm [8], Hochberg [9], Hommel [10] and Dubey/Armitage-Parmar [11] methods. Once the *p*-values have been adjusted, they can be compared to the nominal significance level. For example in the trial on substance abuse [5], two unadjusted *p*-values: 0.010,0.002 were reported. If the Bonferroni method was used, the p-values could have been adjusted to 0.020, 0.004 and compared to the significance level *α* of 0.05. Alternatively, the significance level could be adjusted (to 0.05/2 = 0.025 in this example) and compared to the unadjusted *p*-values.

In clinical trials, it is also important to consider the power of the tests to detect an intervention effect. In the context of multiple outcomes, the power of the study can be defined in a number of ways depending on the clinical objective of the trial: i) ‘disjunctive power’, ii) ‘conjunctive power’ or iii) ‘marginal power’ [12].

The disjunctive power (or minimal power [13]) is the probability of finding at least one true intervention effect across all of the outcomes [12, 14]. The conjunctive power (or maximal power [13]) is the probability of finding a true intervention effect on all outcomes [14]. It may be noted that the disjunctive and conjunctive power have previously been referred to as ‘multiple’ and ‘complete’ power respectively [13]. The marginal (or individual) power is the probability of finding a true intervention effect on a particular outcome and is calculated separately for each outcome. When the clinical objective is to detect an intervention effect for at least one of the outcomes the disjunctive power and marginal power are recommended whereas the conjunctive power is recommended when the clinical objective is to detect an intervention effect on all the outcomes [12, 14]. In this paper, we are focusing on the former clinical objective and therefore we focus on disjunctive and marginal power.

The power requirements of a trial should match the clinical objective which needs to be pre-specified when designing the study and the sample size calculation should be performed accordingly. In current practice, the sample size calculations for trials often focus on the marginal power for each outcome. An approach that has been recommended and is often used in trials is to calculate the sample size separately for each of the primary outcomes by applying a Bonferroni correction to adjust the significance level [15]. The largest value of the sample size is then considered as the final sample size for the trial [16].

Missing outcome data are common in RCTs [17] which will inevitably reduce the power and efficiency of the study [18] which may result in failure to detect true intervention effects as statistically significant.

When using multiple primary outcomes, there is limited guidance as to which method(s) should be used to take account of multiplicity in the sample size calculation and during the statistical analysis.

Some studies have compared a selection of methods which adjust *p*-values to account for multiplicity to handle multiple outcomes in trials. Sankoh, Huque and Dubey [11] compare a selection of adjustment methods for statistical analysis in terms of FWER but they do not evaluate the methods with respect to the power obtained. Blakesley et al. discuss both FWER and power requirements for selected methods for a large number of outcomes with varying degrees of correlation [19]. Lafaye de Micheaux provide formulae to calculate the power and sample size for multiple outcomes [20] which require several assumptions to be made about the outcomes, including normality and whether the covariance matrix between the outcomes is known or not. They discuss global testing procedures, including the Hotelling T^2^ method. None of these studies have investigated the adjustment methods in the presence of missing data.

There is limited literature discussing the sample size requirements for clinical trials with multiple primary outcomes where the clinical objective is to detect an intervention effect for at least one of the outcomes. Dmitrienko, Tamhane and Bretz [14] and Senn and Bretz [13] provide some discussion regarding the sample size in the context of multiple outcomes. However, neither discuss sample size in the context of which adjustment method should be used and they do not provide a comparative table depending on the type of desired power to show implications on the required sample sizes.

In this paper, we compare easy to use methods to adjust *p*-values in terms of FWER and power, when investigating two, three and four outcomes in presence of complete outcome data and outcome data with missing values. We also consider a range of correlations between the outcomes. We consider both marginal and disjunctive power. Based on our findings, we provide practical recommendations on the adjustment methods which could be used for the sample size calculation and analysis of RCTs with multiple primary outcome. We also present tables showing the implications of using the marginal and disjunctive power on the required sample size for a trial under different scenarios.

## Methods

We assume that we have a two-arm trial in which there are *M* primary outcomes. We are interested in testing the null hypotheses *H*_*j*_ (*j* = 1,  … , *M*) that there is no intervention effect on the nominated outcomes. The test statistics *t*_*j*_ are used to test the null hypotheses *H*_*j*_. Further suppose that there is an overall null hypothesis $H(M)={\bigcap}_{j=1}^M{H}_j.$ Under this overall hypothesis, the joint test statistic (*t*_1_,  … , *t*_*M*_) has a M-variate distribution. We denote *p*_*j*_  as the marginal, unadjusted *p*-values obtained from the appropriate statistical test associated with analysing each outcome separately in a univariate framework. For example, when analysing continuous outcomes, an unpaired Student’s t-test may be used or when analysing binary outcomes a Chi-squared test may be used to investigate the intervention. To control the FWER a correction method is then applied to the unadjusted *p*-values (*p*_*j*_). We compare the following commonly used adjustment methods in this paper: Šidák, Bonferroni, Holm, Hochberg and Hommel. In addition, we consider the Dubey/Armitage-Parmar (D/AP) adjustment and Stepdown minP resampling procedure which take account of the pairwise correlation between the outcomes.

The method proposed by Šidák is defined as ${p}_j^{\overset{\check{} }{\mathrm{S}}\mathrm{i}}=1-{\left(1-{p}_j\right)}^M$. Equivalently, the significance level could be adjusted to ${\alpha}^{\overset{\check{} }{\mathrm{S}}\mathrm{i}}=1-{\left(1-\alpha \right)}^{1/M}$, where *α* is the unadjusted significance level. Under the assumption that the outcomes are independent, the adjustment can be derived as

$$ {\displaystyle \begin{array}{c}P\left( no\  Type\kern0.28em I\kern0.28em error\kern0.28em on\kern0.28em \mathbf{1}\kern0.28em test\right)=1-{\alpha}^{\overset{\check{} }{\mathrm{S}}\mathrm{i}},\\ {}\to P\left( no\  Type\kern0.28em I\kern0.28em error\kern0.28em on\kern0.28em \mathbf{M}\kern0.28em test s\right)={\left(1-{\alpha}^{\overset{\check{} }{\mathrm{S}}\mathrm{i}}\right)}^M,\\ {}\to P\left(\boldsymbol{atleast}\  on e\  Type\kern0.28em I\kern0.28em error\kern0.28em on\kern0.28em \mathrm{M}\kern0.28em test s\right)=1-{\left(1-{\alpha}^{\overset{\check{} }{\mathrm{S}}\mathrm{i}}\right)}^M=\alpha .\end{array}} $$

The Bonferroni method is the most common approach to account for multiplicity due to its simplicity. In this method, the unadjusted *p*-values *p*_*j*_ are multiplied by the number of primary outcome =1 −  = 1 −   ≈ s. The Dubey/Armitage-Parmar (D/AP) is an ad-hoc method based on the Šidák method, which takes into account the correlation between the outcomes [11]. The adjusted *p*-value is ${p}_j^{adj}=1-{\left(1-{p}_j\right)}^{g(j)}$ where *g*(*j*) = *M*^1 − *mean ρ*(*j*)^ and *mean ρ*(*j*) is the mean correlation between the *j*^*th*^ outcome and the remaining *M* − 1 outcomes. When using this method in the analysis of multiple outcomes, the mean correlation may be estimated from the data. There has been little theoretical work to assess the performance of this approach [11].One of the nice properties of the D/AP procedure, which may have contributed to its development, is that when the average of the correlation coefficients is zero, the D/AP adjustment is according to the Bonferroni test, and when the average correlation coefficient is one, the D/AP adjusted and the unadjusted *p*-values are the same. The Holm method [8] involves a step-down method, whereby the unadjusted *p*-values are ordered from smallest *p*_(1)_ to largest *p*_(*M*)_ and each unadjusted *p*-value is adjusted as ${p}_{(k)}^{Holm}=\left(M-k+1\right)\ {p}_{(k)}$, where *k* = 1, … *M* is the rank of the corresponding *p*-value. Then starting with the most significant p-value (smallest p-value), each adjusted *p*-value is compared to the nominal significance level, until a p-value *greater* than the significance level is observed after which the method stops [21]. The Hochberg step-up method [9] is similar to the Holm step-down method but works in the other direction. For this method, the unadjusted *p*-values are ranked from largest *p*_(1)_ to smallest *p*_(*M*)_ and adjusted as ${p}_{(k)}^{Hoch}=\left(M-k+1\right)\ {p}_{(k)}$. Starting with the least significant *p*-value (largest *p*-value), each adjusted *p*-value is compared to the pre-specified significance level, until a *p*-value *lower* than the significance level is observed after which the method stops [21]. Contrary to the Šidák based approaches, this is a semiparametric method meaning the FWER is only controlled when the joint distribution of the hypotheses test statistics is known, most commonly multivariate normal [22]. The Hommel method [10] is another data-driven stepwise method. For this method, the unadjusted *p*-values are ranked from largest *p*_(*M*)_ to smallest *p*_(1)_. Then let *l* be the largest integer for which ${p}_{\left(M-l+j\right)}>\frac{j\alpha}{l}$ or all *j* = 1, … *l*. If no such *j* exists then all outcomes can be deemed statistically significant; otherwise, all outcomes with ${p}_i\le \frac{\alpha }{j}$ may be deemed statistically significant, where *j* = 1, … , *M*; *i* = 1, … , *M*. To control the FWER, the Hommel method requires that the joint distribution of the overall hypothesis test statistic is known.

Another step-down method to adjust *p*-values is the ‘Stepdown minP’ procedure [23, 24]. Unlike the previous methods, it does not make any assumptions regarding the distribution of the joint test statistic. Instead it attempts to approximate the true joint distribution by using a resampling approach. This method takes into account the correlation structure between the outcomes and therefore may yield more powerful tests compared to the other adjustment methods [25]. The Stepdown minP adjusted *p*-values are calculated as follows: 1) calculate the observed test statistics using the observed data set; 2) resample the data with replacement within each intervention group to obtain bootstrap resamples, compute the resampled test statistics for each resampled data set and construct the reference distribution using the centred and/or scaled resampled test statistics; 3) calculate the critical value of a level *α* test based on the upper *α* percentile of the reference distribution, or obtain the raw *p*-values by computing the proportion of bootstrapped test statistics that are as extreme or more extreme than the observed test statistic [26]. That is, the Stepdown minP adjusted *p*-value for the *j*^*th*^ outcome is defined as [24, 26] ${p}_j^{minP}={\max}_{k=1,\dots, j}\left\{\kern0.5em \Pr \left(\left(\ {\min}_{l=k,\dots, M}\kern.45em {p}_l\le {p}_k\kern0.5em \right|\kern0.5em H(M)\right)\right\},$ where *p*_*k*_ is the unadjusted *p*-value for the *k*^*th*^ outcome, *p*_*l*_ is the unadjusted *p*-value for the *l*^*th*^ outcome (*l* = *k*, … , *M*), and *H*(*M*) is the overall null hypothesis.

Although, the resampling based methods have previously been recommended for clinical trials with multiple outcomes they are not widely used in practice [25]. The Stepdown minP has been shown to perform well when compared to other resampling procedures [26] and was therefore investigated in this paper.

We perform a simulation study to evaluate the validity of these methods to account for potentially correlated multiple primary outcomes in the analysis and sample size of RCTs. We focus on two, three and four outcomes as a review of trials with multiple primary outcomes in the psychiatry and neurology field found that the majority of the trials had considered two primary outcomes [4]. Additionally, it has been recommended that a trial should have no more than four primary outcomes [27]. We estimate the family wise error rate (FWER), the disjunctive power to detect at least one intervention effect and the marginal power to detect an intervention effect on a nominated outcome in a variety of scenarios.

### Simulation study

We used the following model to simulate values for two continuous outcomes ***Y***_***i***_ = (*Y*_*i*, 1_, *Y*_*i*, 2_),

2 $$ {\boldsymbol{Y}}_{\boldsymbol{i}}={\boldsymbol{\beta}}_{\mathbf{0}}+{\boldsymbol{\beta}}_1{x}_i+{\boldsymbol{\epsilon}}_{\boldsymbol{i}} $$

where *x*_*i*_ indicates whether the participant *i* received intervention or control, ***β***_1_ = ( *β*_11_, *β*_12_ )^*T*^ is vector of the intervention effects for each outcome, ***ϵ***_**i**_  are errors which are realisations of a multivariate normal distribution $$ {\boldsymbol{\epsilon}}_{\boldsymbol{i}}={\left({\epsilon}_{i,1},{\epsilon}_{i,2}\ \right)}^T\sim N\left(\left(\genfrac{}{}{0pt}{}{0}{0}\right),\left(\begin{array}{cc}1& \rho \\ {}\rho & 1\end{array}\right)\ \right), $$ and *ρ ϵ* {0.0, 0.2, 0.4, 0.6, 0.8}. The model was also extended to simulate three and four continuous outcomes. When simulating three and four outcomes we specified compound symmetry, meaning that the correlation between any pair of outcomes is the same. We explored both uniform intervention effect sizes and varying effect sizes across outcomes. For the uniform intervention effect sizes, we specified an effect size of 0.35 for all outcomes, that is ***β***_1_ = (0.35, 0.35)^*T*^, ***β***_1_ = (0.35, 0.35, 0.35)^*T*^ or ***β***_1_ = (0.35, 0.35, 0.35, 0.35)^*T*^ for two, three and four outcomes scenarios respectively. This represents a medium effect size, which reflects the anticipated effect size in many RCTs [28]. For the varying intervention effect sizes, we specified that ***β***_1_ = (0.2, 0.4)^*T*^, ***β***_1_ = (0.2, 0.3, 0.4)^*T*^ or ***β***_1_ = (0.1, 0.2, 0.3, 0.4)^*T*^ for two, three and four outcomes scenarios respectively. We also explored the effect of skewed data by transforming the outcome data with uniform intervention effect sizes to have a gamma distribution with shape parameter = 2 and a scale parameter = 2. The gamma distribution is often used to model healthcare costs in clinical trials [29, 30] and may also be appropriate for skewed clinical outcomes.

We set the sample size to 260 participants, with an equal number of participants assigned to each arm. This provides 80% marginal power to detect a clinically important effect size of 0.35 for each outcome, using an unpaired Student’s t-test and the significance level is unadjusted at 0.05. We introduced missing data under the assumption that the data were missing completely at random (MCAR). When simulating two outcomes, 15 and 25% of the observations in outcome 1 and 2 are missing respectively, and on average approximately 4% of the observations would be missing for both outcomes. When simulating three outcomes, 15% of the observations are missing in one outcome and 25% of the observations are missing in the other two outcomes. When simulating four outcomes, 15% of the observations are missing in two outcomes and 25% of the observations are missing in the other two outcomes. This proportion of missingness in outcomes is often observed in RCTs [31–34].

We estimated the FWER and disjunctive power by specifying no intervention effect (*β*_1*j*_ = 0) and an intervention effect (*β*_1*j*_ ≠ 0), respectively, and calculating the proportion of times an intervention effect was observed on at least one of the outcomes. The marginal power was similarly estimated but we calculated the proportion of times an intervention effect was observed on the nominated outcome. For each scenario we ran 10,000 simulations. The simulations were run using R version 3.4.2. The Stepdown minP procedure was implemented using the NPC package.

We calculated the sample size based on disjunctive power using the R package “mpe” [35] and we calculated the sample size based on the marginal power using the R package “samplesize” [36]. The statistical methodology used for the sample size calculation in these packages is described in the Additional file 1.

## Results

The Bonferroni and Holm methods lead to the same FWER and disjunctive power when analysing multiple primary outcomes. This is because both methods adjust the smallest *p*-value in the same way. Similarly, the Hochberg and Hommel methods lead to same FWER and disjunctive power when two primary outcomes are analysed and differences between these methods arise when analysing three or more outcomes.

### Family wise error rate, FWER

**Fig. 1:**
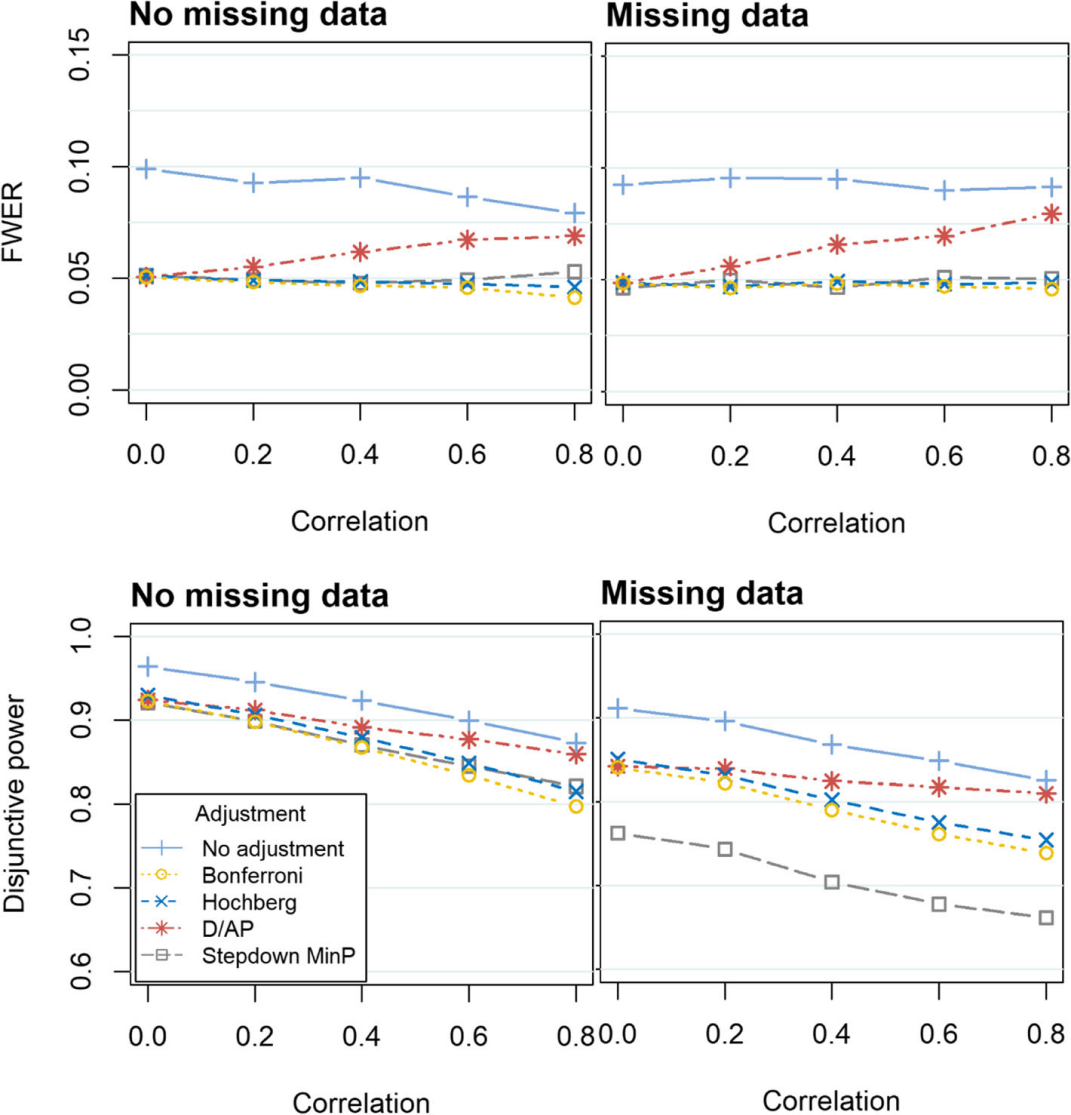
The FWER (top) and disjunctive power (bottom) obtained when evaluating two continuous outcomes using a variety of methods to control the FWER. In the left hand graphs, there are no missing data. In the right hand graphs, the missing data are missing completely at random, with 15% missing in the first outcome and 25% missing in the second outcome (‘Missing data’). The graphs display various degrees of correlation between the outcomes, ranging from *ρ* = 0 to *ρ* = 0.8. The Monte Carlo standard errors (MCSE) were similar across all methods. When there were no missing data, the MCSE was between 0.002–0.004 for the disjunctive power and 0.002–0.004 for the FWER. In the missing data scenario, the MCSE was between 0.002–0.003 for the disjunctive power and between 0.003–0.005 for the FWER.)

**Fig. 2:**
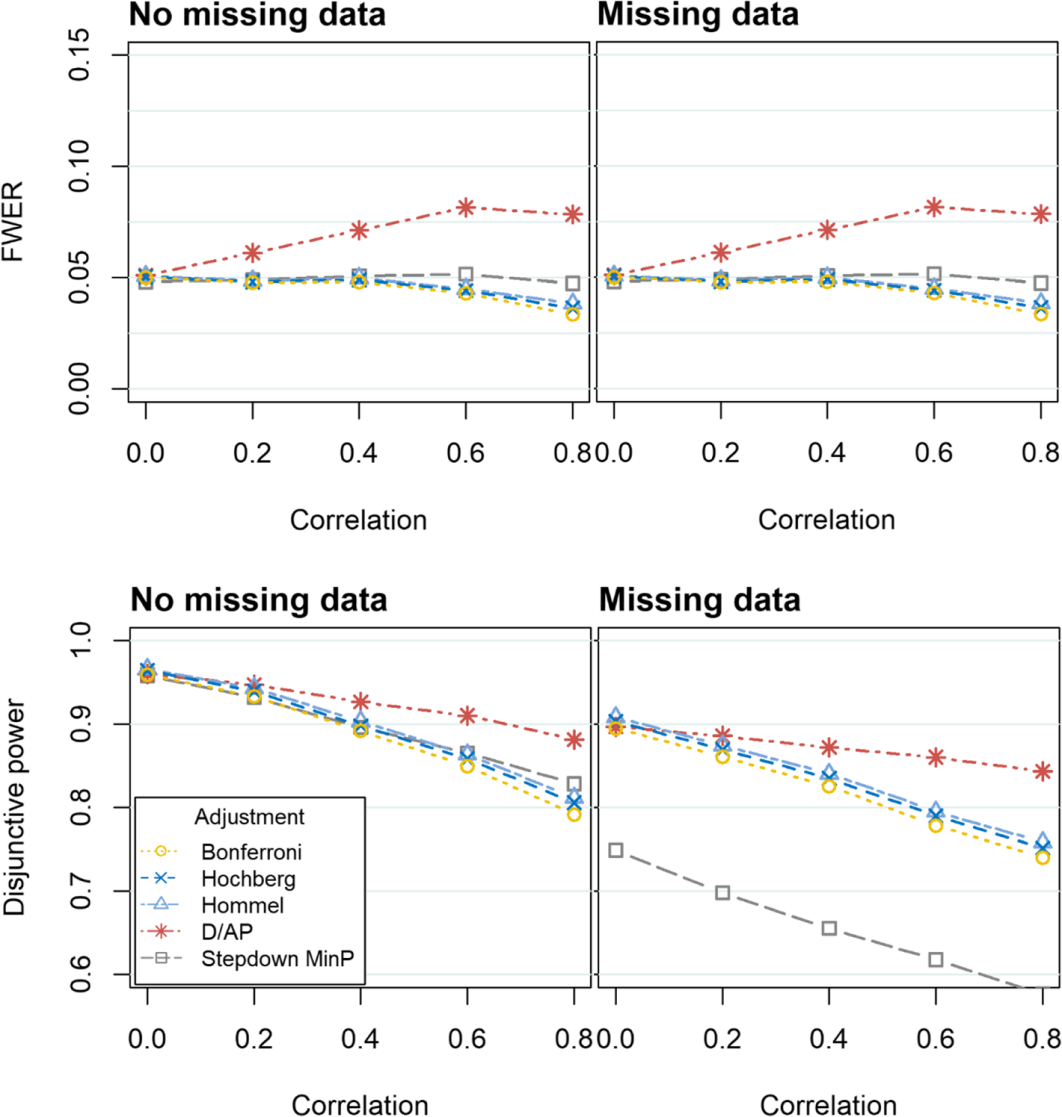
FWER (top) and disjunctive power (bottom) obtained when evaluating three continuous outcomes using a variety of methods to control the FWER. In the left hand graphs, there are no missing data. In the right hand graphs, the missing data are missing completely at random, with 15% missing in one outcome and 25% missing in the other two outcomes (‘Missing data’) The graphs display various degrees of correlation between the outcomes, ranging from *ρ* = 0 to *ρ* = 0.8. The Monte Carlo standard errors (MCSE) were similar across all methods. When there was no missing data, the MCSE was between 0.001–0.004 for the disjunctive power and 0.002–0.004 for the FWER. In the missing data scenario, the MCSE was between 0.001–0.004 for the disjunctive power and between 0.001–0.004 for the FWER

**Fig. 3:**
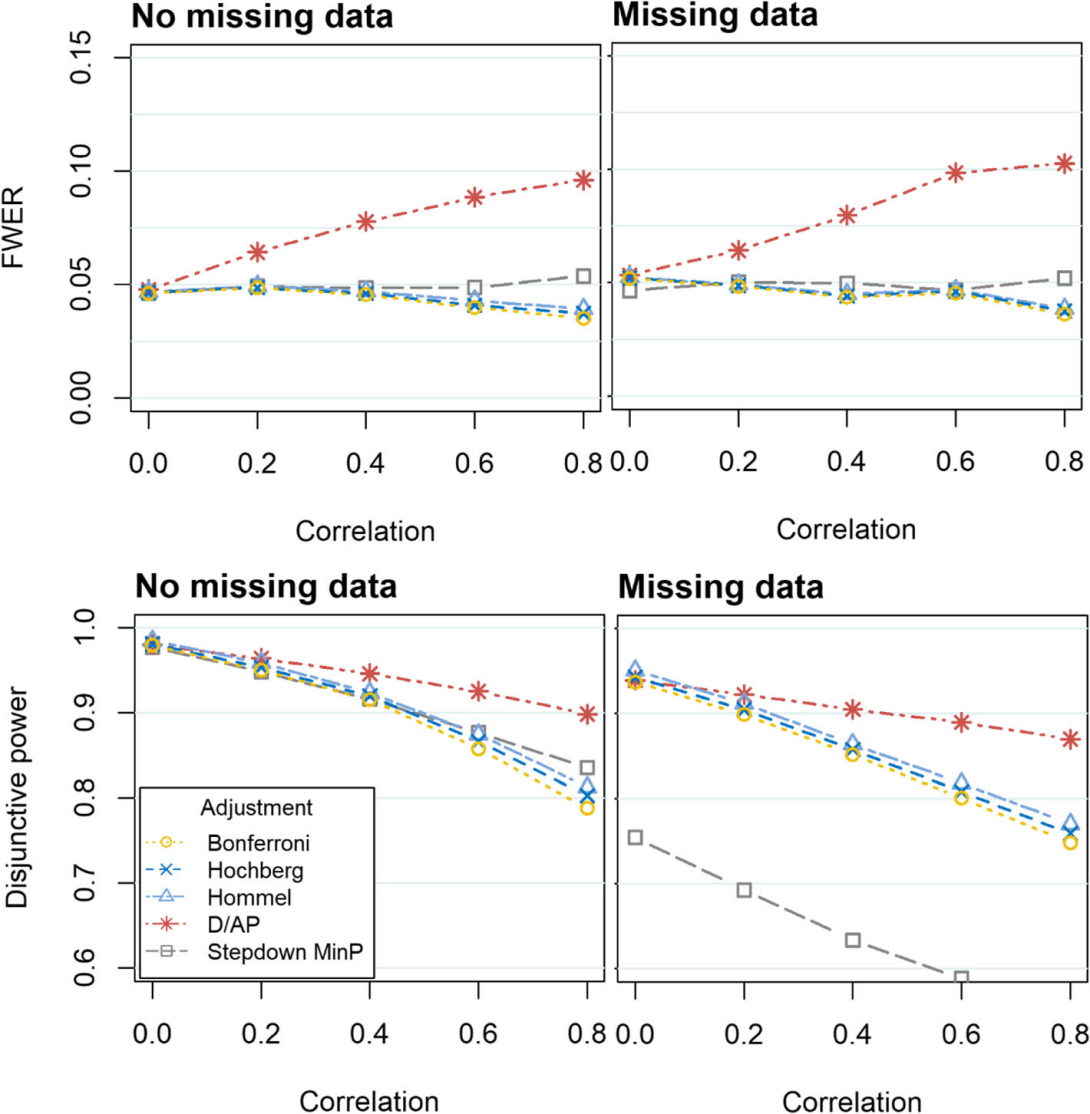
FWER (top) and disjunctive power (bottom) obtained when evaluating four continuous outcomes using a variety of methods to control the FWER. In the left hand graphs, there are no missing data. In the right hand graphs, the missing data are missing completely at random, with 15% missing in two outcomes and 25% missing in the other two outcomes (‘Missing data’). The graphs display various degrees of correlation between the outcomes, ranging from *ρ* = 0 to *ρ* = 0.8. The Monte Carlo standard errors (MCSE) were similar across all methods. When there was no missing data, the MCSE was between 0.001–0.004 for the disjunctive power and 0.002–0.004 for the FWER. In the missing data scenario, the MCSE was between 0.001–0.004 for the disjunctive power and between 0.001–0.004 for the FWER

The FWER obtained when evaluating two, three and four outcomes are displayed in Figs. 1, 2 and 3 respectively. Following on from the explanation above, the Holm and Hommel methods are not displayed in Fig. 1 and the Holm method is not displayed in Fig. 2 or 3. The results for the varying intervention effect sizes and skewed data are presented in the Additional file 1.

When there is correlation between the outcomes (*ρ* ≥ 0.2), the D/AP method does not control the FWER. All other adjustment methods control the FWER in all scenarios. The Stepdown minP performs well in terms of FWER. Unlike the other methods, it maintains the error rate at 0.05 even when the strength of the correlation between the outcomes increases. Differences between the Bonferroni, Hochberg and Hommel methods arise when there is moderate correlation between outcomes (*ρ* ≥ 0.4). The Hommel provides the FWER which is closest to 0.05, whilst being controlled, followed by Hochberg and then Bonferroni. Very similar results were observed when the outcomes followed a skewed distribution, consequently these results are presented in the Additional file 1.

### Disjunctive power

Figures 1, 2 and 3 show that the disjunctive power decreases as the correlation between the outcomes increases for all approaches. We do not consider the power obtained when using the D/AP approach due to its poor performance in controlling the FWER. When there is no missing data, the Stepdown minP and Hommel approaches provide the highest disjunctive power. For weak to moderate correlation (*ρ* = 0.2 *to* 0.6) the Hommel method has slightly more disjunctive power, but the Stepdown minP performs better when there is strong correlation (*ρ* = 0.8). The Stepdown minP procedure gives the lowest power in the presence of missing data. This could be attributed to the fact that it uses listwise deletion removing participants with at least one missing value prior to the analysis thus resulting in a loss of power when there is missing data. As expected the Bonferroni method gives slightly lower power compared to the other methods for complete data but considerably out performs the Stepdown minP method when there is missing data. Very similar results were observed when the outcomes followed a skewed distribution.

When the intervention effect sizes varied, the differences observed between the methods were less pronounced. When using four outcomes with varying effect sizes, very similar disjunctive power were observed to that of constant effect sizes. When using the Hommel adjustment, higher disjunctive power was observed compared to the Holm and Bonferroni methods albeit by a very minimal amount.

### Marginal power

The marginal power obtained for each outcome when using the different adjustment methods are shown in Table 1. In terms of marginal power, the Hommel adjustment was the most powerful method, followed closely by the Hochberg method. When two independent outcomes were analysed, a power of 76.8% was observed after applying a Hommel correction. The power decreased to 76.8 and 75.2% when three and four outcomes were analysed, respectively, after applying a Hommel correction. As expected the Bonferroni method was the most conservative method, providing the least power. However, contrary to popular belief, the Bonferroni method maintains similar levels of power as the strength of correlation increases.

**Table 1:** Marginal (individual) power obtained for each outcome, when analysing two (top), three (middle) or four (bottom) continuous outcomes using a variety of methods to control the FWER

Pairwise correlation between outcomes	None	Bonferroni	Holm	Hochberg	Hommel	Stepdown minP
Two outcomes						
0	80.9	72.4	78.5	79.2	79.2	78.2
0.2	80.6	71.8	77.8	78.6	78.6	77.7
0.4	80.0	71.3	76.6	77.7	77.7	76.7
0.6	80.0	71.0	76.0	77.4	77.4	76.7
0.8	80.3	71.3	75.6	77.4	77.4	77.2
Three outcomes						
0	80.2	65.9	75.2	76.7	76.8	75.5
0.2	80.5	66.4	75.0	76.6	76.7	75.3
0.4	80.2	65.7	73.8	75.4	75.6	73.2
0.6	80.0	65.7	73.3	75.0	75.2	73.8
0.8	80.0	65.9	72.2	74.6	74.8	76.1
Four outcomes						
0	80.5	62.3	73.2	75.0	75.2	72.7
0.2	80.4	62.3	72.6	74.4	74.8	72.2
0.4	80.6	62.4	72.1	74.1	74.4	72.2
0.6	80.3	62.0	70.7	73.1	73.5	72.3
0.8	80.3	61.9	69.7	73.2	73.6	73.5

D/AP method was not examined due to the poor performance observed when exploring FWER

There was no missing data in any of the outcomes. The tables display various degrees of correlation between the outcomes, ranging from no correlation (***ρ =*** **0.0**) to strong correlation (***ρ =*** **0.8**)

When analysing two outcomes the percentage of simulations in which an intervention effect was observed on neither outcome, one outcome or both outcomes are shown in Table 2. When using the Holm method, a statistically significant intervention effect was observed on both outcomes in 48–58% of the simulations. This reduced to 36–48% of the simulations when using the Bonferroni method. As expected, when using the Hochberg adjustment the same results were observed as when using the Hommel adjustment. Compared to Holm, slightly higher percentages of simulations with two statistically significant intervention effects are observed when using Hochberg and Hommel.

**Table 2:** The percentage of simulations in which an intervention effect was observed for neither outcome, one outcome or both outcomes when analysing two outcomes, using a variety of methods to control the FWER

Method	Pairwise correlation between outcomes	Number of outcomes an intervention effect was observed on		
		0	1	2
Bonferroni	0	16.1	48.4	35.5
	0.2	18.6	43.2	38.2
	0.4	20.6	37.7	41.7
	0.6	23.4	32.7	43.9
	0.8	26.3	26.3	47.5
Holm	0	16.1	35.6	48.3
	0.2	18.6	31.0	50.4
	0.4	20.6	26.4	53.0
	0.6	23.4	22.0	54.6
	0.8	26.3	16.0	57.7
Hochberg	0	15.1	35.6	49.4
	0.2	17.6	31.0	51.5
	0.4	19.3	26.4	54.3
	0.6	22.0	22.0	56.0
	0.8	24.8	16.1	59.1
Hommel	0	15.1	35.6	49.4
	0.2	17.6	31.0	51.5
	0.4	19.3	26.4	54.3
	0.6	22.0	22.0	56.0
	0.8	24.8	16.1	59.1
Stepdown minP	0.0	23.7	37.5	38.8
	0.2	25.6	33.6	40.8
	0.4	29.6	27.1	43.4
	0.6	32.2	20.2	47.6
	0.8	33.8	13.8	52.4

In these simulations there was missing data in the outcomes (15% in one outcome and 25% in the other outcome). The tables display various degrees of correlation between the outcomes, ranging from no correlation (***ρ =*** **0.0**) to strong correlation (***ρ =*** **0.8**)

### Sample size calculation

We recommend the Bonferroni adjustment to be used for the sample size calculation when designing trials with multiple correlated outcomes since it can be applied easily by adjusting the significance level and it maintains the FWER to an acceptable level up to a correlation of 0.6 between outcomes. As the Hochberg and Hommel methods are data-driven, it is not clear how these more powerful approaches can be incorporated into the sample size calculation unless prior data are available. Determination of the required sample size using these methods may require simulation-based approach.

In Table 3, we present the required sample sizes to obtain 90% disjunctive power for trials with two outcomes for varying degrees of correlations between the outcomes (*ρ* = {0.2, 0.4, 0.6, 0.8}). For these calculations, we specified that there is equal allocation of participants between the intervention arms. To calculate the sample size a priori information on the degree of correlation between the outcomes is required. More details regarding the sample size calculation are provided in [13]. For comparison, we also present the sample size required to obtain 90% marginal power for each outcome. For all calculations, we have used the Bonferroni method to account for multiple comparisons. We provide the sample sizes required to analyse two, three and four outcomes in Tables 3, 4 and 5, respectively. In Table 5, the top line provides an example sample size calculation for four outcomes where there is a small standardised effect size for all four outcomes (*Δ* = 0.2). When there is weak pairwise correlation between all four outcomes (*ρ* = 0.2), 325 participants would be required into each arm to obtain 90% disjunctive power. As the pairwise correlation increases to *ρ* = 0.8 the required sample size increases to 529. The sample size required to obtain 90% marginal for each outcome in this scenario is 716 participants per trial arm. The number of participants required to obtain 90% marginal power is greater than the number of participants required to obtain 90% disjunctive power. Thus the required sample size varies considerably depending on whether marginal or disjunctive power is used. The smallest of the sample sizes required to obtain the desired marginal power is the required sample size to achieve 90% disjunctive power if the outcomes are perfectly correlated (*ρ* = 1) [37].

**Table 3:** Sample size required to obtain 90% disjunctive power and 90% marginal power when analysing two outcomes, after applying a Bonferroni correction

Standardised effect sizes for each of the 2 outcomes		Sample size required to obtain 90% DISJUNCTIVE power				Sample size required to obtain 90% MARGINAL power for each outcome	
		Correlation between outcomes					
Outcome 1	Outcome 2	0.2	0.4	0.6	0.8	Outcome 1	Outcome 2
0.2	0.2	402	436	475	522	622	622
0.2	0.3	237	251	264	274	622	278
0.2	0.4	145	150	154	156	622	157
0.2	0.5	96	98	99	100	622	101
0.3	0.3	179	194	211	232	278	278
0.3	0.4	126	135	144	152	278	157
0.3	0.5	89	93	97	99	278	101
0.4	0.4	101	109	119	131	157	157
0.4	0.5	78	84	90	96	157	101
0.5	0.5	65	70	76	84	101	101

Sample sizes provided are required per arm. A Bonferroni correction is applied for all calculations to account for the multiple comparisons

**Table 4:** Sample size per group, assuming three outcomes, 90% disjunctive power, after applying a Bonferroni correction

Standardised effect sizes for each of the 3 outcomes			Sample size required to obtain 90% DISJUNCTIVE power				Sample size required to obtain 90% MARGINAL power for each outcome		
			Correlation between outcomes						
Out.^a^ 1	Out. 2	Out. 3	0.2	0.4	0.6	0.8	Out. 1	Out. 2	Out. 3
0.2	0.2	0.2	353	401	456	524	677	677	677
0.2	0.3	0.3	185	207	229	254	677	302	302
0.2	0.4	0.4	109	120	131	143	677	171	171
0.2	0.5	0.5	71	77	84	92	677	110	110
0.3	0.3	0.3	157	179	203	234	302	302	302
0.3	0.4	0.4	101	114	127	143	302	171	171
0.3	0.5	0.5	68	76	83	92	302	110	110
0.4	0.4	0.4	89	101	114	132	171	171	171
0.4	0.5	0.5	64	72	81	91	171	110	110
0.5	0.5	0.5	57	65	73	84	110	110	110

Sample sizes provided are required per arm. A Bonferroni correction is applied for all calculations to account for the multiple comparisons. Key: ^a^‘*Ou*t’ Outcome

**Table 5:** Sample size per group, assuming four outcomes, 90% disjunctive power, after applying a Bonferroni correction

Standardised effect sizes for each of the 4 outcomes				Sample size required to obtain 90% DISJUNCTIVE power				Sample size required to obtain 90% MARGINAL power for each outcome			
				Correlation between outcomes							
Out.^a^ 1	Out. 2	Out. 3	Out. 4	0.2	0.4	0.6	0.8	Out. 1	Out. 2	Out. 3	Out. 4
0.2	0.2	0.2	0.2	325	382	447	529	716	716	716	716
0.2	0.2	0.3	0.3	189	215	242	270	716	716	319	319
0.2	0.2	0.4	0.4	114	127	129	152	716	716	181	181
0.2	0.2	0.5	0.5	75	82	89	98	716	716	116	116
0.3	0.3	0.3	0.3	145	170	199	235	319	319	319	319
0.3	0.3	0.4	0.4	101	117	133	151	319	319	181	181
0.3	0.3	0.5	0.5	71	80	88	98	319	319	116	116
0.4	0.4	0.4	0.4	82	96	112	133	181	181	181	181
0.4	0.4	0.5	0.5	63	73	84	96	181	181	116	116
0.5	0.5	0.5	0.5	52	61	72	85	116	116	116	116

Sample sizes provided are required per arm. A Bonferroni correction is applied for all calculations to account for the multiple comparisons. Key: ^a^‘*Out*’ Outcome

## Discussion

When using multiple primary outcomes in RCTs it is important to control the FWER for confirmatory phase III trials. One approach to do this is to adjust the *p*-values produced by each statistical test for each outcome. Additionally, some of the outcomes are likely to have missing values, consequently this needs to be considered when choosing an appropriate method to adjust the *p*-values.

### Statistical analysis

We found that all methods investigated, except the D/AP, controlled the FWER. This agrees with the results previously reported in [19]. The Stepdown minP performed best in terms of FWER, but the R package used to implement the method uses listwise deletion removing participants with at least one missing value before the analysis resulting in a loss of power. The validity of this approach depends on how the method is implemented and the extent of the missing data.

We recommend that the Hommel method is used to control FWER when the distributional assumptions are met, as it provides slightly more disjunctive power than the Bonferroni and Holm methods. The distributional assumption associated with the Hommel method is not restrictive and is met in many multiplicity problems arising in clinical trials [22]. Even when the data followed a skewed distribution, the Hommel method performed well, showing it may be used to analyse a variety of outcomes, including those with a skewed distribution.

Given the availability of the software packages to implement the more powerful approaches, there is little reason to use the less powerful methods, such as Holm method. For example, the Hommel method can easily be implemented in R or SAS. Even though it is not currently available in Stata or SPSS, the *p*-values can be copied across and adjusted in R. However, if the assumptions cannot be met, the simpler Holm method could be used.

When the intervention effect size varied across the outcomes, we found that the differences in disjunctive power between the methods were less pronounced. It appeared that the outcome with the largest effect size ‘dominated’ the disjunctive power. When the sample size is based on the disjunctive power, the outcomes with the largest effect size would have high marginal power, whereas the outcome with the smallest effect size would have low marginal power – much below the overall desired level of power. It follows that when investigators are looking for an intervention effect for at least one outcome, it is unlikely that they will see an intervention effect on the outcomes with the smaller effect sizes without seeing an intervention effect on the outcomes with the largest effect size. Consequently, in this scenario, it may be advisable to pick the outcome(s) with the largest effect size as the primary outcome(s) and treat the other outcomes as secondary outcomes, however, this decision will need to account for the relative clinical importance of the outcomes. Alternatively, when the intervention effect size varies across the outcomes, investigators may wish to consider ‘alpha spending’ in which the total alpha (usually 0.05) is distributed or ‘spent’ across the M analyses.

We appreciate that in practice the choice of the adjustment method may also depend on other factors, such as the availability of simultaneous confidence intervals and unbiased estimates. It is standard practice to report the 95% confidence intervals alongside point estimates and *p*-values. When using multiple primary outcomes, it may be necessary to adjust the confidence interval so that it corresponds to the *p*-values adjusted for multiplicity. The confidence interval may be easily adjusted when using Bonferroni or Holm adjustments, using the R function “AdjustCIs” in the package “Mediana” [38]. However, it is not straightforward to adjust the confidence interval when using the Hochberg and Hommel. Consequently, the confidence intervals reported may not align with the *p*-values when these adjustments are used. As stated in the European Medical Agency (EMA) guidelines, in this instance, the conclusions should be based on the p-values and not the confidence intervals [3]. If confidence intervals that correspond to the chosen multiplicity adjustment are not available or are difficult to derive, then the EMA guidelines advise that simple but conservative confidence intervals are used, such as those based on Bonferroni correction [3].

The statistical analysis plan of a trial should clearly describe how the outcomes will be tested including which adjustment method, if any, will be used [39].

Our review of trials with multiple outcomes showed that majority of the trials analysed the outcomes separately without any adjustments for multiple comparisons [4]. Where adjustment methods were used, only the most basic methods were used, possibly due to their ease of implementation. The Bonferroni method was the most commonly used method, although the Holm and Hochberg methods were also used. As a consequence, we focused on relatively simple techniques in this paper. However, more advanced approaches, such as graphical methods to control the FWER are available and described in Bretz et al. [40] and Bretz et al. [41] .

It is not necessary to control the FWER for all types of trial designs, for example, for trial designs with co-primary outcomes where all outcomes have to be declared statistically significant for the intervention to be deemed successful. The FDA guidelines state that in this scenario no adjustment needs to be made to control the FWER [39] and the ‘conjunctive’ power is used. We have not evaluated the conjunctive power as it is not relevant to the scenarios considered in this paper. The conjunctive power may be substantially reduced compared to the marginal power for each outcome [39] and is never larger than the marginal power [13]. The conjunctive power behaves in reverse to the disjunctive power in that as the correlation between the outcomes increases, the conjunctive power increases.

Additionally, multiplicity adjustments may not be necessary for early phase drug trials. However, it is generally accepted that adjustments to control the FWER are required in confirmatory studies, that is when the goal of the trial is the definitive proof of a predefined key hypothesis for the final decision making [42].

### Sample size

When designing a clinical trial, it is important to calculate the sample size needed to detect a clinically important intervention effect. Usually the number of participants that can be recruited in a trial is restricted because of ethical, cost and time implications. The sample size calculation for a trial is usually based on an appropriate statistical method which will be used for the primary analysis depending on the study design and objectives. The sample size can vary greatly depending on if the marginal power or overall disjunctive power is used highlighting the importance of calculating the sample size based on the trial objective. To account for multiplicity in the sample size calculation, we recommend that the Bonferroni adjustment is used. The Bonferroni adjustment can be applied easily within the sample size calculation using an analytical formula [39] and our simulation study showed that it maintains the FWER to an acceptable level for low to moderate correlation between the outcomes. Additionally, there is not much loss in power when using the Bonferroni adjustment, compared to the other methods, in the presence of missing data. In contrast, the other methods investigated in this paper are data driven and therefore it is not clear how these can be incorporated without prior data.

One approach that has previously been used to calculate the sample size for multiple primary outcomes, was to calculate the sample size based on the individual marginal powers for each outcome and to choose the maximum sample size for the trial [43]. This approach guarantees adequate marginal power for each individual test. However, this approach will overestimate the number of participants required if the investigators are interested in disjunctive power. Moreover, it may be problematic to achieve that sample size in trials where recruitment is a problem and may result in trials being closed down prematurely. Finally, the sample size should be inflated to account for the expected amount of missing data.

### Study extensions and limitations

In this paper, we only explored continuous outcomes. However, in RCTs binary outcomes or a combination of continuous and binary outcomes may be used. For two binary outcomes, the maximum possible pairwise correlation between the outcomes will be less than one in absolute magnitude [44] and therefore we would expect similar results but with less pronounced differences between methods for the strong correlations.

Additionally, we only explored global effects, that is either no interventions effect on any of the outcomes (*β*_1*j*_ = 0 ) or an intervention effect on all the outcomes (*β*_1*j*_ ≠ 0). Global effects are most realistic when the strength of the correlation between the outcomes is moderate to strong. However, in practice a mixture of no effects and some intervention effects may be observed, especially when the strength of the correlation between the outcomes is weak.

## Conclusions

To ensure that the FWER is controlled when analysing multiple primary outcomes in confirmatory randomised controlled trials, we recommend that the Hommel method is used in the analysis for optimal power, when the distributional assumptions are met. When designing the trial, the sample size should be calculated according to the trial objective. When specifying multiple primary outcomes, if considered appropriate, the disjunctive power could be used, which has smaller sample size requirements compared to that when using the individual marginal powers. The Bonferroni adjustment can be used in the sample size calculation to account for multiplicity.

## Additional file

Additional file 1 Sample size calculation methodology. Varying the effect size across outcomes. Skewed data. (DOCX 1675 kb)

## Data Availability

The datasets analysed during the current study are available from the corresponding author on reasonable request.

## Abbreviations

CI: Confidence interval
D/AP: Dubey/Armitage-Parmar
FWER: Familywise error rate
MCAR: Missing completely at random
SE: Standard error
