# Correction to: Methods to adjust for multiple comparisons in the analysis and sample size calculation of randomised controlled trials with multiple primary outcomes

**DOI:** 10.1186/s12874-019-0807-8

**Published:** 2019-07-22

**Authors:** Victoria Vickerstaff, Rumana Z. Omar, Gareth Ambler

**Affiliations:** 1 Marie Curie Palliative Care Research Department, Division of Psychiatry, University College London, Gower Street, London, WC1E 6BT UK; 2 Department of Statistical Science, University College London, Gower Street, London, WC1E 6BT UK

**Correction: BMC Med Res Methodol (2019) 19:129**

**https://doi.org/10.1186/s12874-019-0754-4**

In the original publication of this article [1], “=1 −  = 1 −   ≈ s” was mistakenly added after the sentence “ In this method, the unadjusted *p*-values pj are multiplied by the number of primary outcome” in the Methods section, and should be deleted.

The original article has been corrected.
