# Supplementary material for: Methods to adjust for multiple comparisons in the analysis and sample size calculation of randomised controlled trials with multiple primary outcomes
Source: BMC Med Res Methodol. 2019 Jun 21;19:129. doi: 10.1186/s12874-019-0754-4 (PMC6588937; doi:10.1186/s12874-019-0754-4)
Supplement: Supplementary file 1 — Sample size calculation methodology. Varying the effect size across outcomes. Skewed data. (DOCX 1675 kb) [file 12874_2019_754_MOESM1_ESM.docx]

**Appendix 1: Sample size calculation methodology**

This appendix contains additional background on the methodology for the sample size calculations described in the method section of the paper. The results described in this section are a concise summary of the relevant results that can be found in textbooks on sample size calculations (Machin et al, 2009; Sozu, 2015).

In all trials, the power requirements should match the clinical objective which should be pre-specified when designing the study and the sample size should be performed accordingly. In current practice sample size calculations often focus on the marginal power for each outcome. However, we may also be interested in the disjunctive power. In this appendix, we describe the sample size calculation required assuming that we are interested initially in maximising the marginal power and secondly the disjunctive power.

We assume that we have a two-arm trial in which there are $M$ primary outcomes. We are interested in testing the null hypotheses $H_{j} (j=1, \ldots, M)$ that there is not an intervention effect on the corresponding outcomes. The test statistics $z_{j}$ are used to test the null hypotheses $H_{j}.$ Further suppose that there is an overall null hypothesis $H_{0}\left( M \right)=\bigcap_{j=1}^{M} H_{j}.$ Under this overall hypothesis, the joint test statistic ${(z_{1},\ldots, z}_{M})$has a M-variate distribution.

**Sample size calculation for marginal power**

We use the marginal power when we are interested in the power to detect an intervention effect on a nominated outcome. The desired marginal power may be specified for each outcome. In this case, we test the hypothesis null $H_{j}$that there is not an intervention effect on the corresponding outcome.

To estimate the sample size we used a unpaired Student’s t-test and we assumed equal variances. Suppose we wish to detect a standardised effect size $\Delta_{j}$, then for significance level $\alpha$, and power $1-\beta$, the number of subjects per group is given by:

$$n =2\frac{2 \left( z_{1-\frac{\alpha}{2}}+z_{1-\beta} \right)^{2}}{\Delta_{j}^{2}}$$

where $z_{1-\alpha/2}$ and $z_{1-\beta}$ are the $\left( 1-\frac{\alpha}{2} \right)$ and $\left( 1-\beta\right)$ quantiles of the standard normal distribution respectively. Further details on the sample size calculation based on the marginal power can be found in the textbook ‘Sample Size Tables for Clinical Studies’ by Machin et al. (2009). In the article, we calculated the required sample size using the R package “samplesize”.

**Sample size calculation for disjunctive power**

We use the disjunctive power when we are interested in testing the overall null hypothesis ($H_{0}(M)$) that there is no difference between intervention groups for all $M$ outcomes. The overall alternative hypothesis ($H_{1}(M)$) is that there is an intervention effect on at least one of the $M$ outcomes. We assume that the variances are known. For the standardised effect size $\Delta_{j}$ for $j= 1,\ldots, M$, the overall disjunctive power is

$$1- \beta=P\left[ \bigcup_{j=1}^{M} \left. \left\{ Z_{j}>z_{1-\frac{\alpha}{2}} \right\} \right| H_{1}(M) \right]$$

$$=1- P\left[ \bigcap_{j=1}^{M} \left. \left\{ Z_{j}\leq z_{1- \frac{\alpha}{2}} \right\} \right| H_{1}(M) \right]$$

$$=1- P\left[ \bigcap_{j=1}^{M} \left. \left\{ Z_{j}^{*}\leq c_{j}^{*} \right\} \right| H_{1}(M) \right]$$

where $Z_{j}^{*}= Z_{j}-\sqrt{jn} \Delta_{j}$ and $c_{j}^{*}=z_{1- \frac{\alpha}{2}}-\sqrt{jn} \Delta_{j}$ and $n$ is the number of subjects per group. The vector of test statistics $(Z_{1}^{*},\ldots, Z_{j}^{*})$ is distributed as an m-variate normal distribution $N_{M}(\boldsymbol{0}, \boldsymbol{\rho}_{\boldsymbol{z}} )$ where the off diagonal element of $\boldsymbol{\rho}_{\boldsymbol{z}}$ is given by $\rho^{jj'}$. The disjunctive power is calculated by using the cumulative distribution function of the m-variate normal distribution. The sample size is the smallest integer required to achieve the desired overall power of 1 − β at the significance level of α. Further details regarding this sample size calculation are provided in a textbook by Sozu et. al (2015).

In the article, we calculated the sample size for a pre-specified disjunctive power using the R package “mpe”, in particular we used the command “atleast.one.endpoint”. The function can be used to computer the sample size for continuous multiple primary outcomes where a significant difference for at least one outcome is expected.

**Appendix 2: Varying the effect size across outcomes**

The following results were obtained by assuming varying intervention effect sizes across continuous outcomes. When analysing two outcomes, we assumed that the intervention effect sizes were 0.2 and 0.4 for the outcomes respectively. When analysing four outcomes, we assumed that the intervention effect sizes were 0.1, 0.2, 0.3 and 0.4 for each of the outcomes respectively.

**Disjunctive power obtained when evaluating two continuous outcomes**

In the left hand graph, there are no missing data. In the right hand graph, the missing data are missing completely at random, with 15% missing in the first outcome and 25% missing in the second outcome (‘Missing data MCAR’).


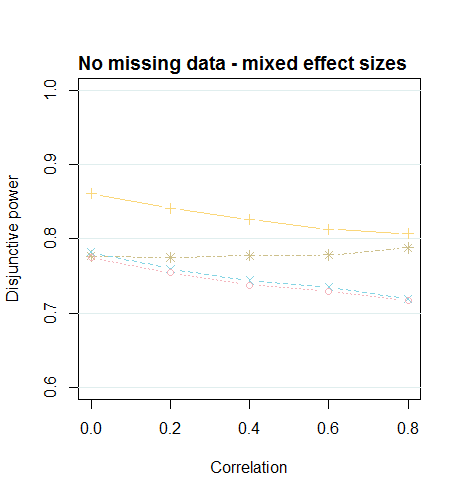

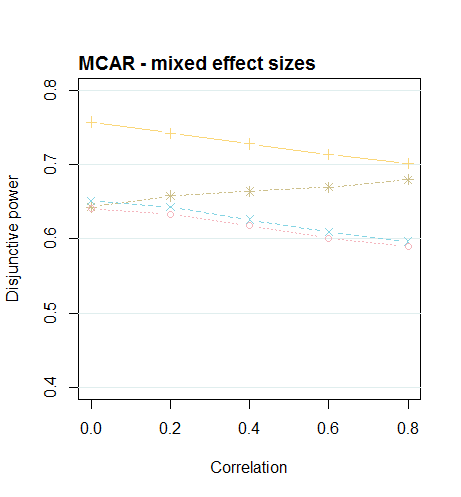


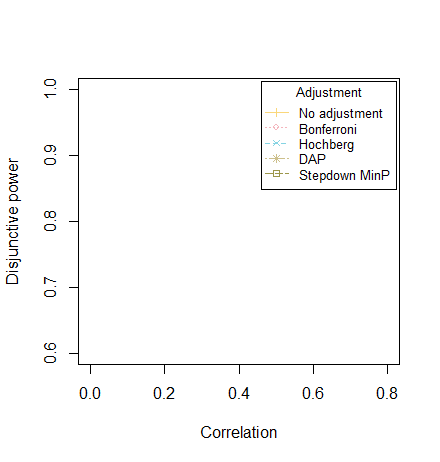


**Disjunctive power obtained when evaluating four continuous outcomes**

In the left hand graph, there are no missing data. In the right hand graph, the missing data are missing completely at random, with 15% missing in two outcomes and 25% missing in the other two outcomes (‘Missing data MCAR’).


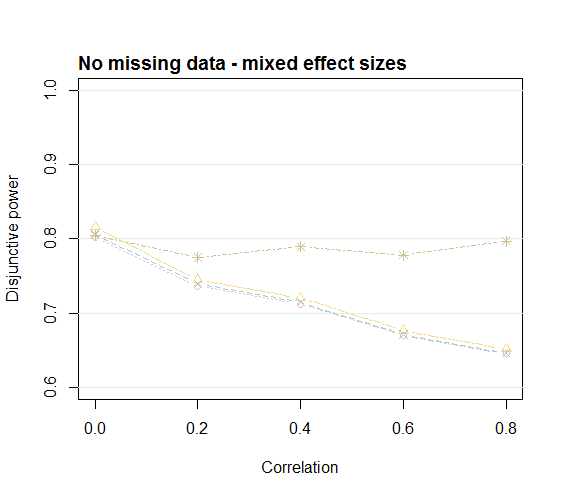

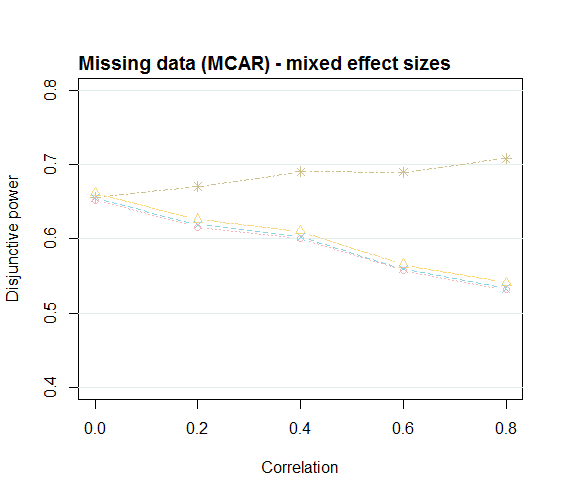


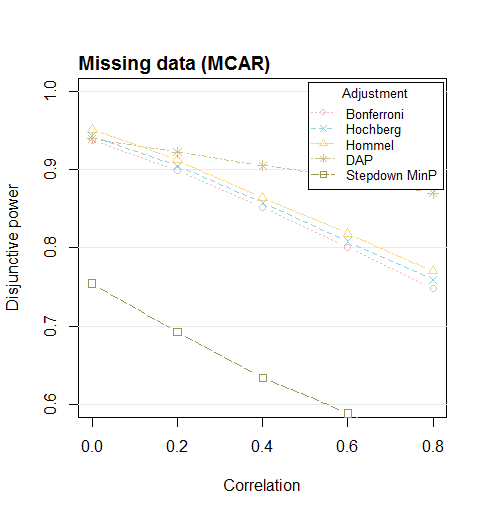


*The simulation standard errors were similar across all methods. When there was no missing data, the simulation standard error was between 0.002-0.004 for the disjunctive power.

**Appendix 3: Skewed data**

We explored skewed outcome data by transforming the data (with uniform intervention effect sizes) to have a gamma distribution with shape parameter = 2 and scale parameter =2. One iteration of the data is shown below to demonstrate the distribution of the data.

**One iteration of the data drawn for outcome 1 and outcome 2**


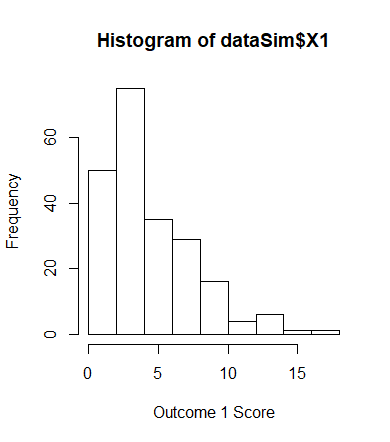

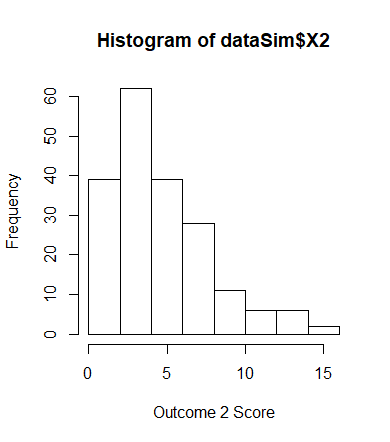


**FWER (top) and disjunctive power (bottom) obtained when evaluating two continuous outcomes which have a skewed distribution**

In the left hand graphs, there are no missing data. In the right hand graphs, the missing data are missing completely at random, with 15% missing in the first outcome and 25% missing in the second outcome.


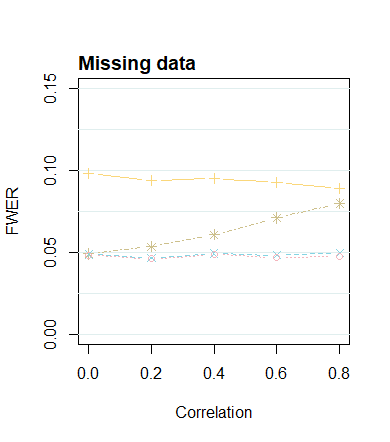

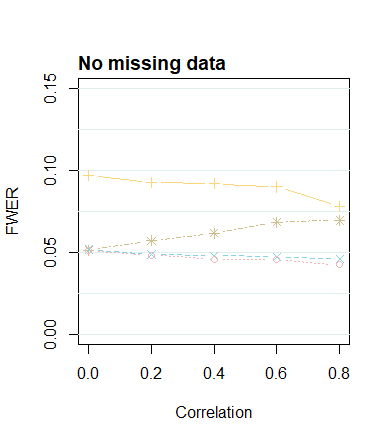

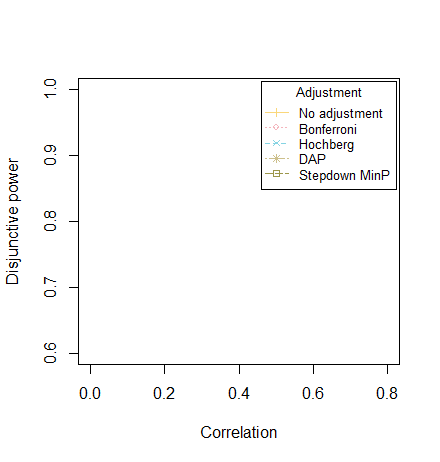

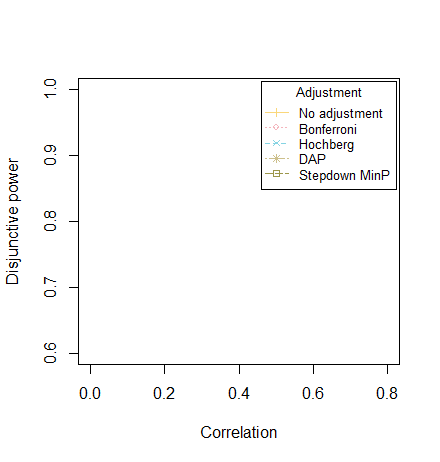

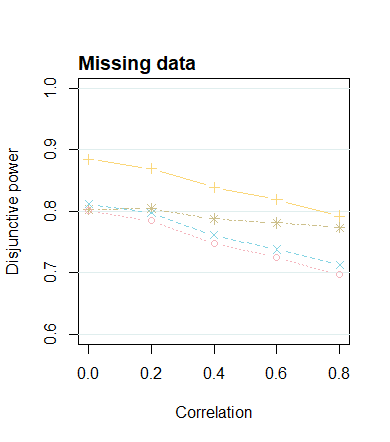

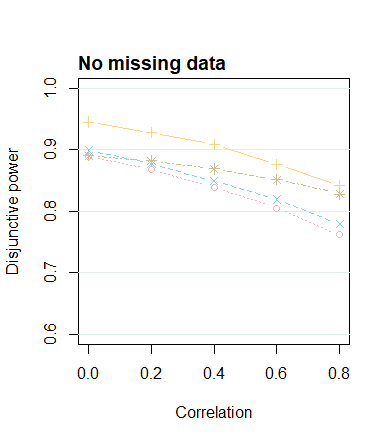


**References**

D. M., M. J. Campbell, S. B. Tan, and S. H. Tan (2009) Sample Size Tables for Clinical Studies, 3rd edition. Oxford: John Wiley & Sons.

Sozu, T. and Sugimoto, T. and Hamasaki, T. and Evans, S.R. (2015). Sample Size Determination in Clinical Trials with Multiple Endpoints. New York: Springer.
